# Vascular development analysis: a study for tertiary anti-vascular endothelial growth factor therapy after second reactivation of retinopathy of prematurity

**DOI:** 10.3389/fmed.2024.1421894

**Published:** 2024-07-19

**Authors:** Xuerui Zhang, Jie Peng, Yuan Yang, Yongqing Liu, Wenting Zhang, Victoria Y. Gu, Huanyu Liu, Haodong Xiao, Jiawei Yin, Yu Xu, Peiquan Zhao

**Affiliations:** ^1^Department of Ophthalmology, Xin Hua Hospital Affiliated to Shanghai Jiao Tong University School of Medicine, Shanghai, China; ^2^Shanghai Jiao Tong University School of Medicine, Shanghai, China; ^3^Department of Ophthalmology, Shandong Second Provincial General Hospital, Jinan, China; ^4^Johns Hopkins Bloomberg School of Public Health, Baltimore, MD, United States

**Keywords:** anti-vascular endothelial growth factor (anti-VEGF), tertiary anti-VEGF therapy, reactivation, vascular development analysis, second reactivation, retinopathy of prematurity (ROP)

## Abstract

**Purpose:**

To observe the vascular development results of tertiary anti-vascular endothelial growth factor (anti-VEGF) therapy following spontaneous second reactivation of retinopathy of prematurity (ROP).

**Methods:**

This retrospective study included 22 infants (42 eyes) with Type 1 or aggressive ROP (A-ROP) who received three anti-VEGF drug treatments for ROP from January 2018 to December 2022. The vascular growth, possible associated risk factors, and the retinal vascularization (DB/DF ratio) were assessed.

**Results:**

The mean follow-up was 17.6 months. After the 3^rd^ intravitreal injection, seven eyes showed complete vascularization (Group 1), while the remaining 35 eyes demonstrated persistent avascular retina (PAR) (Group 2). In Group 2, 17 eyes maintained a stable state and were classified in the regression subgroup. The other 18 eyes developed a 3^rd^ reactivation (reactivation subgroup) and were treated with laser photocoagulation (LPC).

Birth weight (BW) was significantly lower in Group 2 than in Group 1 (*p* < 0.001). The decision tree analysis shows that only infants weighing more than 1,250 g (17.50%) had a chance to achieve complete retinal vascularization. The possibility of PAR was higher in patients with BW <1,250 g than ≥1,250 g (70.00% vs. 12.50%). In addition, most infants with BW ≥ 1,290 g and initial ROP disease in Zone I or posterior Zone II developed PAR.

**Conclusion:**

Tertiary IVR can successfully treat a second ROP reactivation and improve peripheral retinal vascularization. BW is the most significant factor related to complete retinal vascularization. Our decision tree model may be helpful in predicting the prognosis of anti-VEGF drugs in the event of a second ROP reactivation.

## 1 Introduction

Retinopathy of prematurity (ROP) represents a significant cause of visual impairment and potential blindness among premature infants worldwide, posing substantial challenges for pediatric healthcare (1). In recent years, the main treatment method for ROP has shifted from cryotherapy to laser photocoagulation (LPC), and now primary injection of an anti-vascular endothelial growth factor (anti-VEGF) with or without delayed laser therapy (2–5). The benefits of anti-VEGF therapy, such as ease of administration, rapid response, and preservation of peripheral vision, have been widely documented. Additionally, anti-VEGF treatment is associated with a lower incidence of high myopia compared to laser therapy (6–8). However, anti-VEGF agents have been associated with more angiographic abnormalities, such as persistent avascular retina (PAR) and a higher reactivation rate, compared to laser therapy (9, 10).

Several studies have investigated the rates and timing of ROP reactivation and the risk factors associated with ROP reactivation following anti-VEGF monotherapy (11, 12). There is currently no consensus on the treatment of reactivated ROP after initial anti-VEGF therapy. Some suggest LPC or another anti-VEGF drug may be used to treat ROP reactivation (13, 14), while others advocate repeated anti-VEGF therapy combined with LPC (12, 15). Recently, a proposal was conducted for the choice of retreated modality that was based on the reactivation characteristics (16). Here, anti-VEGF therapy was applied for flat vessels, and anti-VEGF therapy combined with LPC was applied for neovascularization. Both these therapies have been applied in our previous clinial practice. Our team has demonstrated that ROP reactivations can be treated successfully with repeated anti-VEGF therapy (17). LPC was selected in cases with financial constraints or those with difficulty following-up (4). However, to our knowledge, no studies have explored the efficacy of anti-VEGF therapy for the second reactivation of ROP, and there is currently no treatment consensus.

Several prior studies have suggested that complete vascular outgrowth can be achieved after one anti-VEGF therapy (18, 19). Moreover, our team has identified that complete retinal vascularization can be achieved after a 2^nd^ anti-VEGF treatment (17). In general clinical opinion, complete retinal vascularization is considered the ideal result of anti-VEGF therapy for ROP. To the best of our knowledge, no study has investigated the factors associated with complete retinal vascularization after anti-VEGF therapy and the ideal treatment for the 2^nd^ ROP reactivation. This study aimed to evaluate the efficacy of tertiary intravitreal administration of an anti-VEGF agent to treat a second ROP reactivation, to describe its effect on retinal vascularization promotion, and to assess possible risk factors indicating a poor prognosis following the third anti-VEGF therapy.

## 2 Method

### 2.1 Study design

This retrospective study was approved by the Ethics Committee of Xinhua Hospital, affiliated with the Shanghai Jiao Tong University of Medicine (XHEC-D-2022-222) and adhered to the Declaration of Helsinki. The study reviewed 1, 140 ROP infants who received anti-VEGF therapy from January 2018 to December 2022, enrolling 22 ROP infants (42 eyes) who received three treatments of anti-VEGF drugs. All patients were routinely followed up for a minimum of 6 months after the 3^rd^ treatment. Written informed consent was obtained from the guardian of each participant.

### 2.2 Diagnosis and treatment

Diagnosis and classification of ROP were based on the International Classification of Retinopathy of Prematurity, Third Edition (ICROP3) (20). All patients underwent a fundus examination with Retcam III Imaging System (Clarity Medical System, Pleasanton, CA) at every visit. The indications for intravitreal administration of an anti-VEGF agent were Type 1 ROP or aggressive ROP (A-ROP) (20, 21). An intravitreal injection of 0.25 mg/0.025 mL Ranibizumab (Lucentis; Novartis, Basel, Switzerland) (IVR) was given with a 30-gauge needle 1.0 mm to the posterior of the limbus under topical anesthesia.

Follow-up appointments were conducted on the following day, as well as 1 week, 4 weeks, and 8 weeks after the intravitreal injection, and every 8 weeks if there was no ROP reactivation. In the case of ROP reactivation, a second intravitreal injection was given; ROP reactivation was defined as the reappearance of retinal abnormalities such as ridge and plus disease (20). The follow-up schedule was the same as that of the first intravitreal injection. Then, the third intravitreal injection was applied when the second reactivation occurred, following the same follow-up schedule as the first injection. If a third reactivation occurred, fluorescein angiography (FFA) and LPC were performed. All intravitreal injections and laser treatments were performed by the same experienced surgeon (P.Q.Z.).

### 2.3 Classification of patients and data collection

**Figure 1:**
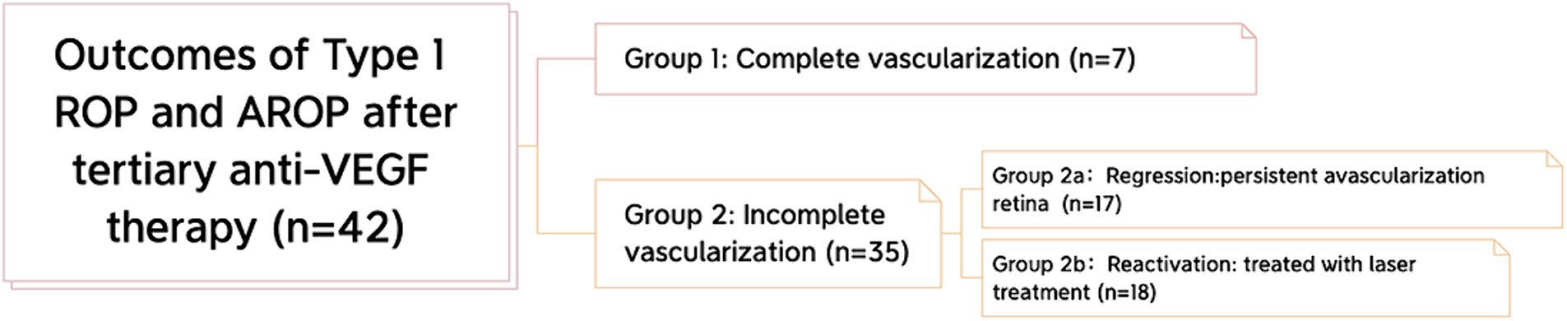
Classification of the outcomes after tertiary anti-VEGF therapy. All eyes were classified into 2 groups: complete vascularization and incomplete vascularization. Furthermore, in the incomplete vascularization group, we classified infants of ROP into 2 subgroups: the regression subgroup and the reactivation subgroup.

All ROP eyes were classified into two groups according to the extent of their retinal vascularization: ROP infants with complete retinal vascularization (Group 1) and ROP infants with incomplete vascularization (Group 2) (Figure 1). Complete retinal vascularization was characterized by a measured distance of less than 2 optic disc diameters (DDs) between the boundary of retinal vascularization and the ora serrata of the temporal side (9). Group 2 was further divided into two subgroups: the regression group (Group 2a), characterized by the presence of PAR but no vascular activity, such as retinal exudation or vascular dilation, and the reactivation group, which requiresd further laser treatment (Group 2b). After mydriasis, retinal vascularization was evaluated by indirect binocular ophthalmoscopy with scleral indentation when needed.

**Figure 2:**
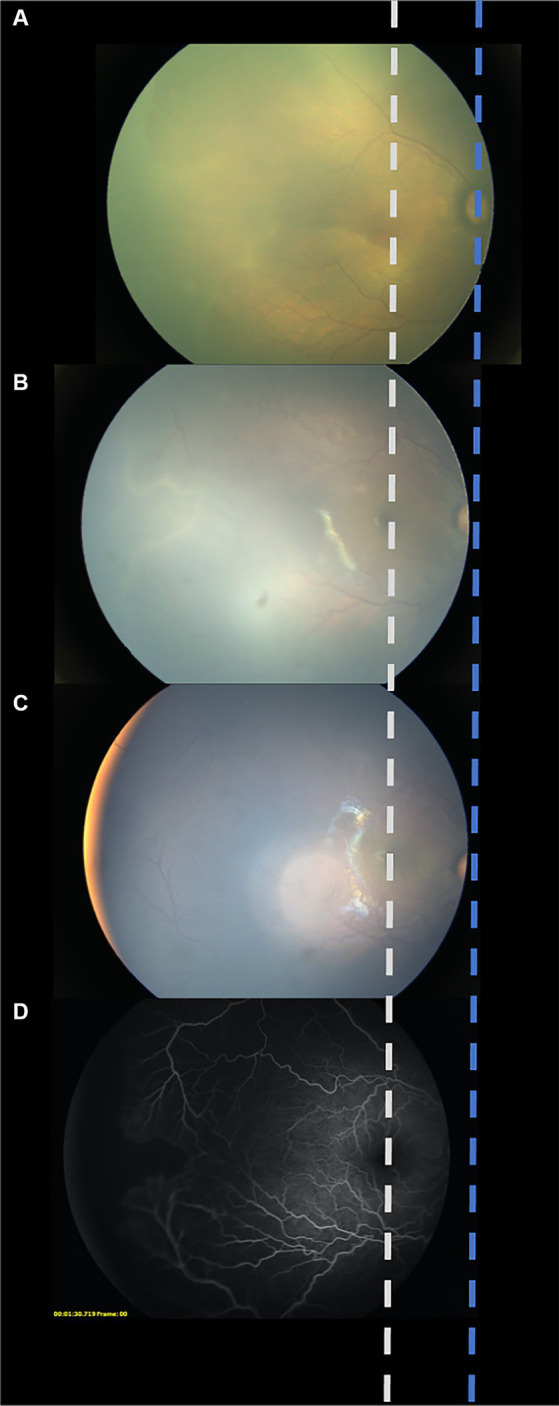
Schematic diagram created from aligning the center of the optic disc and the macula in every photo. To avoid manual measurement errors, fundus photographs were taken from the same position at every treatment and at the final visit were aligned and compiled into one diagram. Fundus photograph before the first IVR **(A)**. Fundus photograph before the second IVR **(B)**. Fundus photograph before the third IVR **(C)**. Fundus photograph of fluorescein angiography after a third reactivation **(D)**.

The following parameters were recorded: sex, birth weight (BW), gestational age (GA), initial ROP characteristics (zone and stage before the first treatment), postmenstrual age (PMA) at every treatment (weeks) and follow-up appointment, the interval between each treatment, retinal hemorrhage, other ocular complications, and systemic conditions. The ratio of the distance from the center of the optic disc to the boundary of the vascularized region (DB) to the distance from the center of the disc to the fovea (DF) (DB/DF) was used to quantify the degree of vascular growth at every treatment and the final visit (18). Fundus photographs were taken from the same position at every treatment and at the final visit to avoid manual measurement errors. Fundus photographs before the first IVR (A), before the second IVR (B), before the third IVR (C), and/or FFA after a third reactivation (D) were compiled into one diagram, and the center of the optic disc and the macula of every photo were aligned (Figure 2). To sufficiently show the far periphery, the foveal reflection and optic disc were at times not shown in the same image; a corresponding dash line was used to represent the estimated location of the macula and optic disc, increasing the precision of the measurement. The DB/DF value was measured individually by three blinded researchers and averaged.

### 2.4 Statistical analysis

Statistical analysis was performed using SPSS version 26.0 (SPSS, Chicago, IL, United States) and R version 4.1.0. Descriptive statistics were used to present the demographic and clinical characteristics of the study population. Means and standard deviations (mean ± SD) were calculated for numerical variables. Furthermore, an unpaired student t-test was used to compare the differences in numerical variables, and categorical variables were presented as frequency (%) and compared using chi-squared tests. Clustlasso, pCOR and random Forest R package were used to predict the possibility of complete retinal vascularization. The possible associated risk factors of ROP reactivation were also analyzed. In addition, a decision tree was analyzed by the rpart R package. A *p-value* < 0.05 was considered statistically significant.

## 3 Results

A total of 42 eyes of 22 infants (two unilateral cases) were included in this study. Among them, 23 eyes (54.76%) demonstrated acute ROP or in zone I. All baseline characteristics are listed in Table 1. The mean BW and GA were 1130.00 ± 232.25 grams and 27.62 ± 1.39 weeks, respectively. The mean follow-up was 17.60 ± 0.60 months. The PMA for the three IVR injections was 34.15 ± 1.61, 42.68 ± 3.18, and 51.92 ± 4.29 weeks. Furthermore, the DB/DF ratios at the three intravitreal injections were 2.51 ± 0.60, 3.44 ± 0.41, and 3.85 ± 0.33, and the mean intervals between the 1^st^ and 2^nd^ injections, and the 2^nd^ and 3^rd^ injections were 7.50 ± 3.02 and 10.55 ± 3.71 weeks, respectively.

**Table 1:** General information and clinical characteristics (*n* = 42 eyes).

Variable	All eyes	Group 1	Group 2	*p* value
	(*n* = 42)	(*n* = 7)	(*n* = 35)	
Male gender, no. (%)	24 (57.14%)	5 (71.43%)	19 (54.29%)	0.502
BW(g), Mean ± SD	1130.00 ± 232.25	1331.43 ± 88.40	1081.38 ± 230.56	<0.001*
GA (weeks), Mean ± SD	27.62 ± 1.39	28.14 ± 0.90	27.3 ± 1.51	0.167
DB/DF at the first IVR, Mean ± SD	2.51 ± 0.60	2.39 ± 1.17	2.55 ± 0.47	0.772
PMA at the first IVR (weeks), Mean ± SD	34.15 ± 1.61	33.29 ± 1.25	34.33 ± 1.63	0.119
Interval before the first reactivation (weeks), Mean ± SD	7.50 ± 3.02	7.00 ± 1.53	7.61 ± 3.27	0.465
DB/DF at the second IVR, Mean ± SD	3.44 ± 0.41	3.49 ± 0.41	3.43 ± 0.42	0.750
PMA at the second IVR (weeks), Mean ± SD	42.68 ± 3.18	40.29 ± 0.76	42.00 ± 3.29	0.013*
Interval before the second reactivation (weeks), Mean ± SD	10.55 ± 3.71	13.57 ± 3.95	9.80 ± 3.39	0.014*
DB/DF at the third IVR, Mean ± SD	3.85 ± 0.33	4.05 ± 0.15	3.80 ± 0.34	0.008*
PMA at the third IVR (weeks), Mean ± SD	51.92 ± 4.29	53.86 ± 3.63	51.47 ± 4.35	0.188
ROP characteristics at initial IVR, no. (%)				
A-ROP and zone I	23 (54.76%)	3 (42.86%)	20 (57.14%)	0.355
Posterior II 2 +	14 (33.33%)	2 (28.57%)	11 (31.43%)	
II 2 +	3 (7.14%)	2 (28.57%)	1 (2. 86%)	
II 3 +	2 (4.76%)	0	2 (5.71%)	
Retinal hemorrhage at initial IVR, no. (%)				0.981
No	29 (69.05%)	5 (71.43%)	24 (68.57%)	
Yes	13 (30.95)	2 (28.0.57%)	11 (31.43%)	
Major neonatal comorbidities, no. (%)				0.075
No	26 (61.90%)	2 (28.0.57%)	24 (68.57%)	
Yes	16 (38.10%)	5 (71.43%)	11 (31.43%)	

BW, birth weight; SD, standard deviation; GA, gestational age; PMA, postmenstrual age; DB/DF, the ratio of the distance from the center of the optic disc to the boundary of the vascularized region (DB) to the distance from the center of the disc to the fovea (DF); IVR, an intravitreal injection with ranibizumab; ROP, retinopathy of prematurity; A-ROP, aggressive ROP. *p<0.05.

After the 3^rd^ injection, seven eyes showed complete vascularization (Group 1), while the remaining 35 eyes had PAR (Group 2). In Group 2, 17 (48.60%) eyes remained in a stable state PAR without vascular activity, such as retinal exudation or vascular dilation, and were classified in the regression subgroup (Group 2a).

The DB/DF at the final visit in Group 2a was 4.24 ± 0.33. The other 18 eyes (51.40%) constituted Group 2b and were treated by LPC after the 3^rd^ reactivation or when vascular leakage was observed (Figure 1).

BW in Group 2 was significantly lower than in Group 1 (*p* < 0.001). Moreover, a significantly higher PMA at the 2^nd^ IVR (*p* = 0.013) and a shorter interval before the 2^nd^ reactivation (*p* = 0.014) were observed in Group 2 compared to Group 1. In addition, the DB/DF ratio was significantly lower in Group 2 than in Group 1 (*p* = 0.006). All reactivations or active diseases were resolved at the last visit without severe adverse anatomic outcomes such as macular ectopia, dragged disc, or retinal detachment.

**Figure 3:**
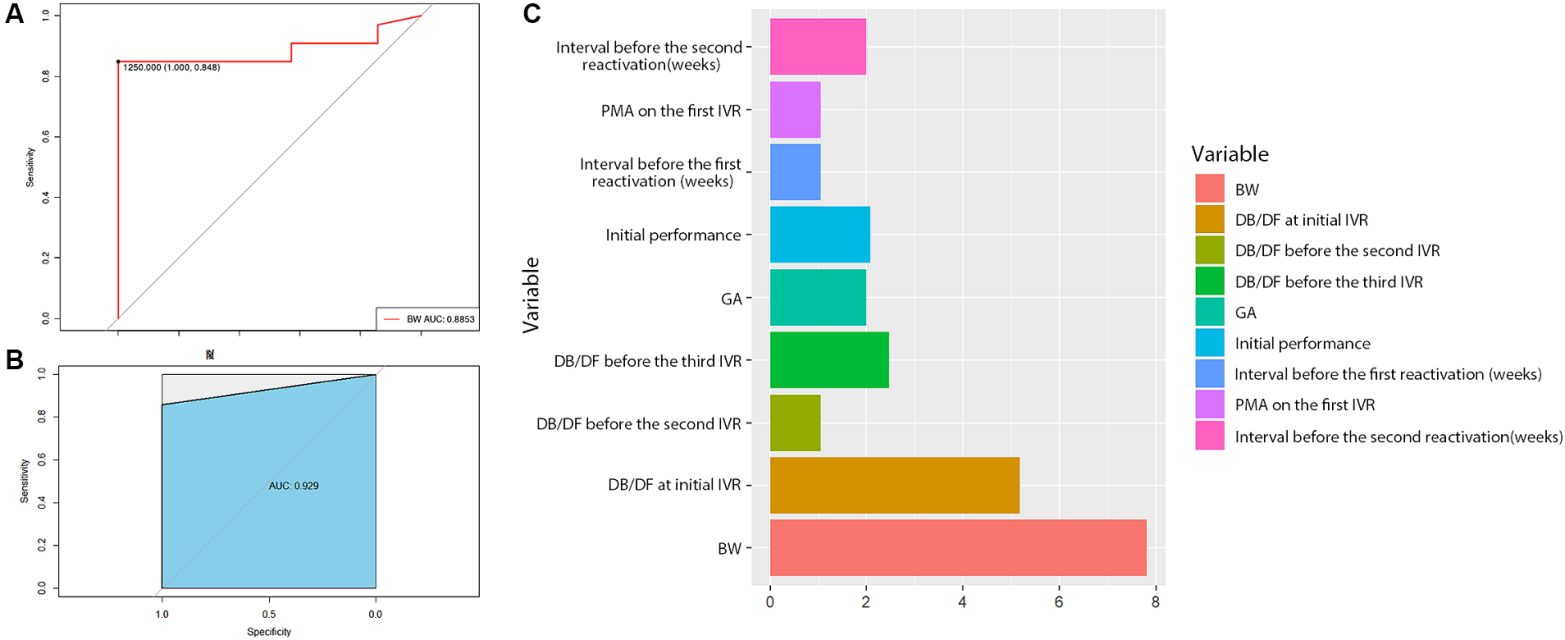
Statistical results of the baseline characteristics. The results suggested that BW = 1,250 g was a cut-off value for complete retinal vascularization **(A)**, and the random forest model further demonstrated the predictive role of BW **(B)**. In addition, the importance analysis by the rpart R package also showed that BW was the most important factor **(C)**.

To explore possible predictors for complete retinal vascularization after the 3^rd^ IVR, the baseline characteristics in Table 1 were analyzed, revealing that BW was the most related predictor, with a cut-off of 1,250 g. The results indicated that ROP patients with BW > 1,250 g were more likely to develop complete retinal vascularization. The AUC value was 0.89 (Figure 3A), and the random forest model (AUC = 0.93) further supported the predictive effect of BW (Figure 3B). Additionally, importance analysis by the rpart R package also suggested that BW was the most important factor (Figure 3C).

**Figure 4:**
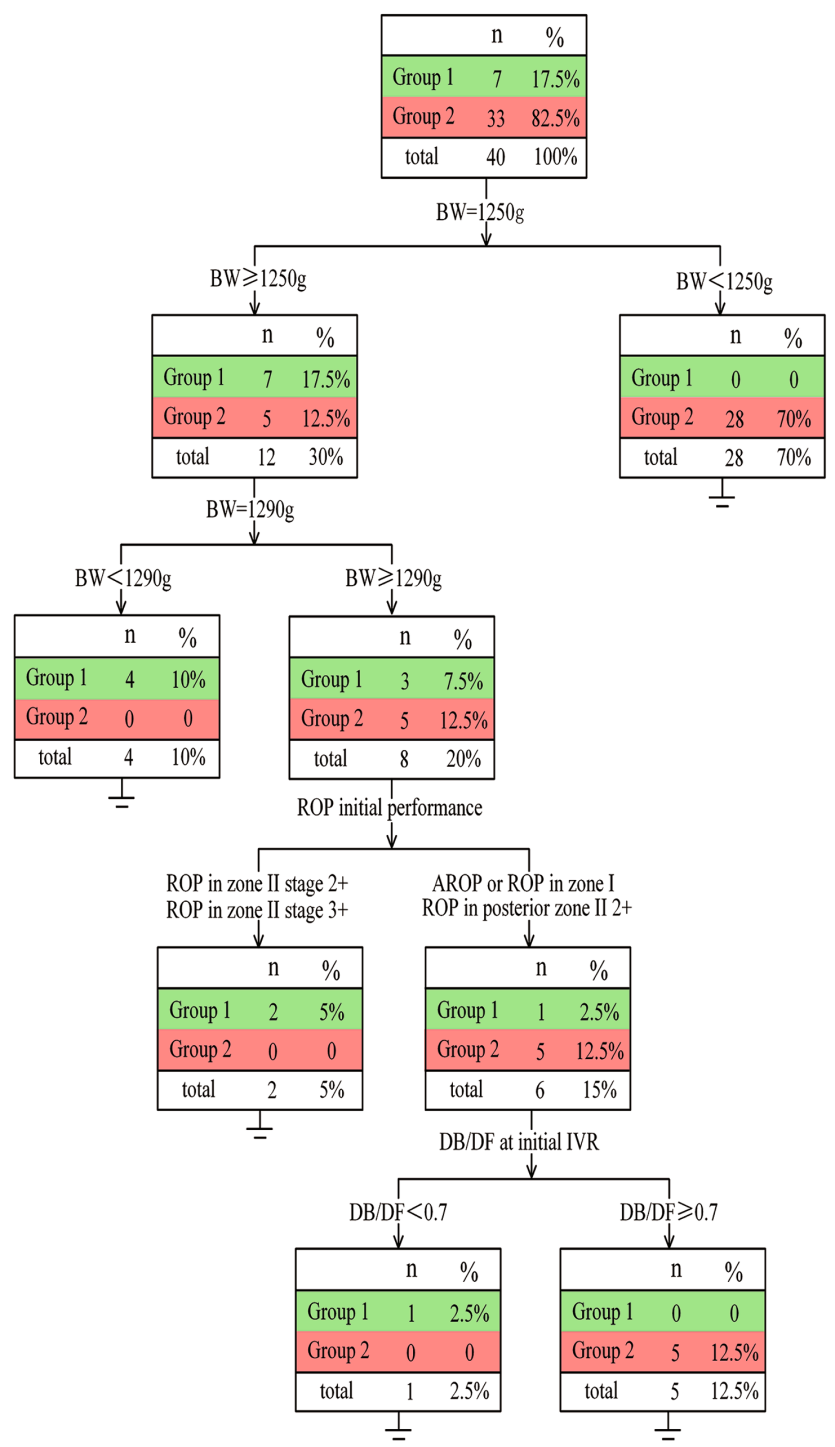
Details of the decision tree It has been showed that for infants of ROP with BW ≥ 1,250 g, their possibility of complete vascularization after tertiary IVR is higher than that of those with BW < 1,250 g (17.5% vs. 0). Moreover, infants with BW > 1,290 g and ROP in zone I or posterior II or A-ROP exhibited a lower rate of complete retinal vascularization (2.5% vs. 5%).

Based on the above findings, a decision tree model was generated by the rpart R package to predict the anatomic outcomes of tertiary IVR (Figure 4). A total of 40 eyes were eligible to build the decision tree model (the other two eyes were excluded due to insufficient information). The decision tree revealed that only when infants with BW higher than 1,250 g had a chance for complete retinal vascularization (17.50%). ROP infants with BW > 1,290 g tended to exhibit complete retinal vascularization (5.00% vs. 0%) if the initial ROP disease was in Zone II (excluding posterior Zone II). However, ROP infants with BW > 1,290 g and initial ROP disease in Zone I or posterior Zone II more often developed PAR instead of complete retinal vascularization (12.50% vs. 2.50%).

Of particular interest, among the six eyes with BW > 1,290 g who had ROP in zone I or posterior Zone II, only one infant with initial DB/DF less than 0.70 developed complete vascularization (2.50% vs. 12.50%).

## 4 Discussion

This is the first known study to describe the effect of tertiary intravitreal anti-VEGF injections following a second reactivation. This study is also the first to construct a decision tree model to predict the extent of retinal vascularization. The decision tree model revealed that the BW, ROP initial characteristics, and DB/DF before the first IVR were related to the extent of retinal vascularization. To our knowledge, this is the first study to investigate the factors associated with tertiary IVR required for complete retinal vascularization.

The publication of BEAT-ROP study has contributed major insights into anti-VEGF therapy for severe ROP. Anti-VEGF therapy has several distinct advantages over LPC. However, several studies have reported a higher incidence of reactivation (especially with IVR therapy) and more vascular changes with anti-VEGF therapy than with laser therapy (12, 22). Prior to establishing the ICROP3 guidelines, there was no clear consensus on the definition of reactivation after anti-VEGF therapy. Therefore, the assessment of reactivation and the timing of reactivation treatment depended on individual experience across different regions. Currently, the ICROP3 guidelines have clarified the definition of reactivation, pre-plus and plus, which made our assessments more precise (20). Despite the risk of PAR after anti-VEGF therapy (23, 24), our group has previously demonstrated that repeated IVR can still be effective in treating the reactivation of ROP and can promotes further vascularization and reduces the area of PAR (17).

In this study, the interval between the initial anti-VEGF therapy and the first reactivation was 7.50 ± 3.02 weeks, which was shorter than in previous studies. For example, Huang et al. reported an interval of 8.30 ± 2.70 weeks, Lyu et al. reported a peak at 8.00 weeks, and Liang et al. reported an interval of 7.87 ± 0.65 weeks in Zone I ROP and A-ROP and 8.40 ± 0.88 weeks in Zone II ROP (4, 11, 14). This finding may be attributed to the fact that more than half of the eyes (23 eyes, 54.76%) were classified as A-ROP or ROP in zone I.

The PMA at the first IVR was similar in Groups 1 and 2 (33.29 ± 1.25 vs. 34.33 ± 1.63), indicating comparable baseline demographics across these groups. In comparison, Wu et al. reported a higher PMA of 36.2 ± 2.7 weeks, and Ling et al. reported a PMA of 36.0 ± 2.34 weeks. This indicates that our study’s baseline PMA is earlier than those reported in the previous studies (4, 12). This may because that 54.76% of eyes in this study had ROP in zone I or A-ROP, which may contribute to disease severity and require earlier and multiple treatments with a poorer prognosis than in other studies. Nevertheless, no significant difference in the PMA at the initial injection, the interval before the first reactivation, and the PMA at the third injection was found between the two groups. However, PMA at the second IVR was significantly smaller in Group 1 than in Group 2 (40.29 ± 0.76 vs. 42.00 ± 3.29, *p* = 0.013), which may indicate that the earlier 2^nd^ anti-VEGF treatment may elicit better vascular development and predict better retinal vascularization.

In addition, a statistically significant difference was found in DB/DF at the third IVR between Group 1 and Group 2 (4.05 ± 0.15 vs. 3.80 ± 0.34, *p* = 0.008). At the third injection, the retina with a smaller avascular zone is more likely to develop complete retinal vascularization and have a better prognosis. Moreover, the interval before the second reactivation was significantly longer in Group 1 than in Group 2 (13.57 ± 3.95 vs. 9.80 ± 3.39 weeks, *p* = 0.01). VEGF concentration in the vitreous cavity is associated with the size of the avascular (ischemic) retina (25). Group 1 exhibited a smaller avascular retinal area than Group 2, which may result in lower and slower VEGF release, leading to delayed reactivation in Group 1.

Despite the success of anti-VEGF therapy for treating ROP, concerns have arisen over the limited number of infants reaching complete retinal vascularization even with treatment. Our study included 42 eyes, and only seven eyes (16.70%) achieved complete retinal vascularization (Group 1). These results indicate that tertiary IVR treatment is effective for a second ROP reactivation, providing a chance for complete retinal vascularization and better anatomical outcomes. However, this conclusion remains controversial, as there is much debate about whether PAR should be treated. According to the guidelines of ICROP3, PAR is prone to cause thinning, holes or lattice-like changes in the retina. Moreover, it may be associated with a higher risk of retinal detachment (20). In our previous practice, LPC was applied only in case of vascular leakage. In the present study, 17 eyes (40.50%) with PAR did not receive any additional treatment, and all were stable at the last visit. Meanwhile, 18 eyes (42.80%) were treated with LPC due to vascular leakage or a 3^rd^ reactivation. Further studies with extended follow-up time is necessary as adolescents and adults are also at risk for reactivation of ROP (26).

This study investigated the effect of tertiary IVR and highlighted concerns regarding the extent of retinal vascularization. Based on this, a decision tree was made to identify the factors associated with tertiary IVR for complete retinal vascularization. GA and BW played a predictive role in the development of ROP and constituted the primary basis for developing the ROP screening criteria (27). Similar to previous studies on this subject, our study demonstrated that BW was the best predictor. Complete retinal vascularization was more likely achieved in ROP infants with BW over 1,250 g. For ROP infants with BW < 1,250 g, higher rates of incomplete retinal vascularization with PAR or further treatment after tertiary IVR were observed compared to those with BW ≥ 1,250 g. Moreover, infants with BW > 1,290 g and ROP in zone I or posterior II or A-ROP also exhibited a lower rate of complete retinal vascularization (2.50% vs. 5.00%). A total of 6 infants with BW > 1,290 g were diagnosed with ROP in zone I or ROP in posterior II or A-ROP, but only one achieved complete retinal vascularization. Therefore, if the second reactivation of ROP occurs, LPC should be recommended instead of anti-VEGF for the patients whose BW is less than 1,250 g due to the low chance of complete retinal vascularization. Furthermore, the application of the third anti-VEGF therapy should be carefully considered for infants with BW exceeding 1,290 g with an initial ROP in zone I or posterior zone II.

Nevertheless, the inherent limitations of the current retrospective study should be acknowledged. Additionally, the size of the cohort was small, and the follow-up duration was relatively short, so the long-term neurodevelopmental outcomes after repeated anti-VEGF therapy could not be determined. A randomized prospective study should be designed to support the results of this study and the effectiveness of the decision tree. Moreover, retinal vascularization (DB/DF value) was assessed using fundus photographs rather than FFA images, which is a subjective method and less precise. Some factors might interfere with the assessment, including the spherical shape of the eyeball, retinal hemorrhage, and immature macular development. To mitigate measurement errors and improve the reliability of the results, the photos were compiled, the macula and the disc were aligned. Third, some factors may not have been included in generating the decision tree, and further studies with more factors are needed.

In conclusion, tertiary IVR is effective in treating a second ROP reactivation and promotes further peripheral retinal vascularization. BW is the factor most related to complete retinal vascularization. Our decision tree model may be helpful for clinicians to evaluate the application of anti-VEGF drugs in a second ROP reactivation.

## Data availability statement

The raw data supporting the conclusions of this article will be made available by the authors, without undue reservation.

## Ethics statement

The studies involving humans were approved by the Ethics Committee of Xinhua Hospital, affiliated with the Shanghai Jiao Tong University of Medicine (XHEC-D-2022-222). The studies were conducted in accordance with the local legislation and institutional requirements. Written informed consent for participation was not required from the participants or the participants' legal guardians/next of kin in accordance with the national legislation and institutional requirements. Written informed consent was obtained from the minor(s)' legal guardian/next of kin for the publication of any potentially identifiable images or data included in this article.

## Author contributions

XZ: Writing – original draft, Writing – review & editing, Conceptualization, Data curation, Formal analysis. JP: Data curation, Funding acquisition, Writing – review & editing, Conceptualization. YY: Data curation, Formal analysis, Investigation, Writing – original draft, Methodology. YL: Writing – review & editing, Methodology. WZ: Data curation, Formal analysis, Writing – original draft. VG: Writing – review & editing, Visualization, Data curation, Formal analysis. HL: Writing – review & editing, Data curation, Formal analysis. HX: Formal analysis, Writing – review & editing, Data curation. JY: Data curation, Formal analysis, Writing – review & editing. YX: Supervision, Writing – review & editing. PZ: Supervision, Writing – review & editing, Funding acquisition.
